# Translation and cross-cultural adaptation of the European Health Literacy Survey Questionnaire, HLS-EU-Q16: the Icelandic version

**DOI:** 10.1186/s12889-020-8162-6

**Published:** 2020-01-14

**Authors:** Sonja Stelly Gustafsdottir, Arun K. Sigurdardottir, Solveig A. Arnadottir, Gudmundur T. Heimisson, Lena Mårtensson

**Affiliations:** 1 Faculty of Health Science, University of Akureyri, Solborg, Nordurslod 2, 600 Akureyri, Iceland; 2 Akureyri Hospital, Eyrarlandsvegi, 600 Akureyri, Iceland; 3 Department of Physical Therapy, Faculty of Medicine, University of Iceland, Saemundargotu 2, 102, Reykjavik, Iceland; 4 Faculty of Psychology, University of Akureyri, Solborg, Nordurslod 2, 600 Akureyri, Iceland; 5 Health and Rehabilitation at Institute of Neuroscience and Physiology, University of Gothenburg, 455, Arvid Wallgrens Backe, hus 2, 405 30 Gothenburg, Sweden

**Keywords:** Health literacy, HLS-EU-Q16, Translation and adaptation, Cognitive interviewing, Validation, Instrument

## Abstract

**Background:**

Health literacy (HL) is defined as the knowledge and competences of people to meet the complex demands of health in modern society. It is an important factor in ensuring positive health outcomes, yet Iceland is one of many countries with limited knowledge of HL and no valid HL measurement. The aim of this study was to translate the European Health Literacy Survey Questionnaire- short version (HLS-EU-Q16) into Icelandic, adapt the version, explore its psychometric properties and establish preliminary norms.

**Methods:**

The HLS-EU-Q16 translation model included three steps: 1) translation-back-translation of HLS-EU-Q16 including specialists’ review (*n* = 6); 2) cognitive interviewing of lay people (*n* = 17); and 3) psychometric analysis with survey participants. The HLS-EU-Q16 includes 16 items, with scores ranges from zero (low/no HL) to 16 (high HL). Statistics included were descriptive, internal consistency measured by Cronbach’s *α*, exploratory factor analysis, and multivariate linear regression.

**Results:**

After the translation and cognitive interviewing, 11 of the HLS-EU-Q16 items were reworded to adapt the instrument to Icelandic culture while maintaining their conceptual objectives. Survey participants were 251. Internal consistency of the translated and adapted instrument was *α* = .88. Four factors with eigenvalues > 1.0 explained 62.6% of variance. Principal component analysis with Oblimin rotation presented four latent constructs, “Processing and Using Information from the Doctor” (4 items, *α =* .77), “Processing and Using Information from the Family and Media” (4 items, *α =* .85), “Processing Information in Connection to Healthy Lifestyle” (5 items, *α* = .76), and “Finding Information about Health Problems/Illnesses” (3 items, α = .73). Lower self-rated health was an independent predictor of lower HL (*β* = −.484, *p* = .008). Preliminary norms for HL ranged from five to 16 (M 13.7, SD ± 2.6) with 72.5% with sufficient HL (score 13–16), 22% with problematic HL (score 9–12) and 5.5% with inadequate HL (score 0–8).

**Conclusions:**

The Icelandic version of HLS-EU-Q16 is psychometrically sound, with reasonably clear factor structure, and comparable to the original model. This opens possibilities to study HL in Iceland and compare the results internationally. The translation model introduced might be helpful for other countries where information on HL is missing based on lack of validated tools.

## Background

Health literacy (HL), as a construct, was introduced within public health research almost 45 years ago [1] and since that time has become an increasingly relevant issue for global public health [2]. It has a broad and inclusive definition referring to “personal characteristics and social resources needed for individuals and communities to access, understand, appraise and use information and services to make decisions about health, or to have implications on health” [3]. HL represents a strong connection to social determinants of health and has been recognised as an important factor in ensuring positive health outcomes [4]. Although high HL does not entail empowerment [5], it is argued that that HL is critical to empowerment of people [6]. Based on this there is a need to improve public access to health information and people’s capacity to use it effectively.

Whereas HL has been researched for decades in native English-speaking countries, the field is still in its early stages in non-English speaking parts of Europe and is only marginally integrated in health research, policy and practice [7, 8]. However, HL gained relevance on the European Commission’s (EC) health agenda [9], and a working group, called the European Health Literacy (HLS-EU) Consortium, was established [10]. Based on content analysis, the HLS-EU Consortium developed an integrated conceptual model, capturing comprehensive evidence-based dimensions of HL, with definition of HL as “… people’s knowledge, motivation and competences to access, understand, appraise and apply health information …” [11]. The design and developmental process of the European Health Literacy Survey Questionnaire (HLS-EU-Q) was described in Sørensen et al. [10]. The Consortium’s work resulted in four versions of the questionnaire: [1] the core version (HLS-EU-Q47) [2]; the extended version Q86 (including additional 39 items relating to selected HL determinants and outcomes) [3]; the short version Q16 [12]; and short-short version Q6 [13]. Using the HLS-EU-Q86, in a survey of HL in eight countries in Europe (*N* = 8000), results indicated that subgroups within the population, defined by financial deprivation, low social status, low education, or old age, had higher proportions of people with limited HL [14]. The results demonstrated that the questionnaire was useful for identifying strengths and weaknesses in HL levels, both within and between countries [14]. In a critical review of population HL assessments, it was noted that the HLS-EU-Q differs from most others HL assessment tools as it is designed to measure HL of general populations rather than specific patient groups. However, an acknowledged limitation of the HLS-EU-Q is a continued emphasis on healthcare and disease prevention and less on health promotion [15].

The number of self-report questionnaires has increased rapidly [16] as has the growth in adapting health status measures to other languages and cultures [17]. Due to Iceland’s population of only 350,000 people, approximately [18], assessment tools are not commonly developed specifically for Icelandic circumstances. A more common approach is to translate and adapt foreign tools to the Icelandic context with a translation-back-translation procedure or with a specialist’s review [19]. Both methods have been criticised, mostly for leaving out the process where lay people respond to the instrument [20]. The term Cross-Cultural Adaptation (CCA) describes the process of viewing both language and cultural issues in preparing a questionnaire for use in another setting [17, 21]. A review of 31 different CCA methods [16] demonstrated that many different recommendations do exist, but a formal standard has not been established, and universal consensus on all aspects of the process has not been reached. As HL has been defined in an inclusive way, meaning it is more than transmitting information and is related to empowerment of lay people, the translation and adaptation of HL instruments should emphasise participation by the people they are intended for. As a part of the cross-cultural adaptation procedure, cognitive interviews are becoming increasingly important [20]. Cognitive interviews are methods to identify problematic survey items by asking research participants to report what is going through their mind, either during or after responding to the survey. The technique helps analyse the manner in which respondents understand, mentally process, and ultimately respond to the presented materials [20, 22]. The cognitive interview technique is reported to be useful when translating questionnaires to other languages [23], and the validity of the technique has been supported when identifying linguistic problems in the questionnaire’s items [24].

There is limited knowledge on HL in Iceland and no validated instruments are available. The increasing interest in HL in the general population worldwide and across Europe supports the demand for HL measurements in Icelandic. Thus, the aim of our study is to translate the European Health Literacy Survey Questionnaire- short version (HLS-EU-Q16) into Icelandic, adapt the version, explore its psychometric properties and establish preliminary norms.

## Methods

### Study design and setting

The HLS-EU-Q16 translation model included three basic steps. In step one, the questionnaire was translated and back- translated with a specialists’ committee review. In step two, 17 lay people responded to the questionnaire in cognitive interviews. In step three, the final Icelandic version of the questionnaire (HLS-EU-Q16-IS) was validated in a stratified random sample, drawn from the Icelandic national registry, including 11 background questions added by the researchers. This same general sample of Icelandic adults was used to establish preliminary norms for health literacy based on HLS-EU-Q16-IS. A permission for the translation was obtained from the HLS-EU-Q project leader and the Icelandic Data Protection Authority was informed about the study.

### Instrument

The HLS-EU-Q16 is one of four questionnaires that resulted from analysis of HLS-EU-Q data from a large, cross-national survey of EU citizens using Eurobarometer methodology [10, 12] where data was collected by using either computer-assisted personal interviews or paper-assisted personal interviews [14]. The original HLS-EU-Q item selection was guided by a conceptual model of HL, which identifies four competencies related to managing health information (find, understand, appraise, and apply) in three domains (health care, disease prevention, and health promotion). These four competencies in three domains were used to create a four by three HL-EU matrix, including 12 cells with unique content for HL [10, 12, 14]. The 16 items in the HLS-EU-Q16 questionnaire were selected as they represent well 11 out of the 12 cells in this HL-EU matrix and at the same time present good psychometric properties. The construct validity of this 16-item scale has been established in all eight HLS-EU study participating countries, based on Rasch modelling for content- and face validity criteria [25]. In Italy e.g. computer-assisted telephone interviews were used in the validation process of the Q16 version [26]. All four HLS-EU-Q questionnaires use the same four response categories for each item. However, when scoring the HLS-EU-Q16, the categories; “very difficult”, “fairly difficult”, “fairly easy” and “very easy” are dichotomized into “easy” (scored with 1) and “difficult” (scored with 0). Summing these responses gives a HLS-EU-Q16 final score that can range from 0 (low/no HL) to 16 (high HL). Missing responses are replaced with 0, given that no more than two responses are missing. For interpretation of the final score on the scale, three levels have been defined: Inadequate HL (0–8), Problematic HL [9–12], and Sufficient HL [13–16] [13, 25]. The HLS-EU-Q16 was selected for translation and adaptation because it is short, easy to administer and is one of few HL instruments designed to measure HL of general populations rather than specific patient groups.

### Participants and sampling method

For the first step of the research, four people were selected for the translation of the questionnaire and six people to create a specialist committee to review translations of the questionnaire twice in the process. Two of those participating in the translations came from the specialists committee and two were hired from outside because of their experience in health related translations. Members of the specialist committee were selected based on their field of specialty to create a multi-disciplinary group within health and social sciences.

To access lay people, for the cognitive interviewing in the second step of this research, administrators of two public institutions, each with 70–80 employers, were contacted in January and February 2017. The institutions were chosen, as they were not in the field of health-related service but in education and social welfare, and were known to have employers with various educational levels. The administrators forwarded, by e-mail, an introduction letter provided by the researchers to their respective employees with general information about the study and information about the right not to participate or to withdraw from the study at any time. Those willing to participate were asked to contact the administrator who forwarded the information on to the researchers. Initially, only one round was planned with 12 people. Because of new information received after the first round, it was decided to add another round. For the first round, 15 were willing to participate and 12 were selected according to the criteria for equal gender, age groups (18–45, 46–64, and 65–85) and educational level distribution (elementary, secondary or university degree). For the second round, five were willing to participate and were selected.

Participants in the validation process (step three) were a part of a stratified random sample of 1200 Icelanders, drawn from the Icelandic National Registry. The sample was stratified according to age, gender and place of living. Inclusion criteria was to be between 18 and 85 years old and registered with a home address in Iceland. People living in nursing homes were excluded. Due to name and address not matching, 91 questionnaires were returned. Therefore, the intended sample size was 1109.

### The translation model

#### Translation of the instrument

The translation and adaptation process are described in Table 1. The original validated English version of the HLS-EU-Q16 was translated into Icelandic by authors 1 and 2, based on recommendations from Beaton et al. [17]. The two authors are fluent (which includes knowing colloquial phrases, jargon, idiomatic expressions, etc.) in the source language, and the target language is their native tongue. Both are familiar with source and target cultures, both have lived and studied in an English speaking country, and both have some knowledge in the content of the instrument. After discussion between the researchers, and with a review from the specialists’ committee, the two versions were combined into one Icelandic version that was translated back into English by two translators. The two back translators are native speakers of the source language and are fluent in the target language. Both back translators have lived and worked in both source and target cultures but are unfamiliar with the content of the instrument. The English back-translated versions were combined into one by the researchers and compared with the original HLS-EU-Q16. Minor discrepancies were addressed based on consensus. Based on these differences, another consultation with the specialists´ committee took place before the final version, for the cognitive interview process, was made.

**Table 1:** Translation and adaptation process of the instrument

Original HLS-EU-Q16 English version	
STEP 1 TRANSLATION	
Forward translation: April 2016 Two translators, fluent in both source and target language with the target language as their native tongue, familiar with both cultures, and having some knowledge in the content of the instrument made two independent forward translations, *versions T-1 and T-2.*	
Integration I: April 2016 Versions T-1 and T-2 were merged into a single version by authors 1 and 2, *version T-1.2*.	
Specialists´ committee review I: May 2016 The multi-disciplinary specialist committee consisted of six members with a PhD in health or social sciences at two public universities in Iceland and most were experienced researchers. Review of the committee reflected on translations and critical decisions. Authors 1 and 2 reached a consensus on any discrepancy and *versions T-1.2.1* was made.	
Back translation: June–July 2016 Two translators, fluent in both source and target language with the source language as their native tongue, familiar with both cultures but unfamiliar with the content of the instrument made two independent back translations, working with T-1.2.1 version.	
Integration II: August 2016 Two independent back translations were merged into a single *version T-2* by authors 1 and 2.	
Specialists´ committee review II: December 2016 Review of all translations by the specialists’ committee and critical decisions. Authors 1 and 2 made a *Pre-final version T-2.1* to be used for cognitive interviewing.	
STEP 2 PRE-TESTING I	
Cognitive interviewing I: January–February 2017 Twelve interviews taken by author 1 and a research assistant (RA) with the pre-final version T-2.1. Comments made on nine questions.	
Consultation and integration I: February 2017 Summary of all item comments reviewed. Authors 1, 2 and RA made *version T-2.2*.	
Cognitive interviewing II: February 2017 Five interviews conducted by RA with people that volunteered participation with the pre-final version T-2.2. Comments made on five questions.	
Consultation and integration II: February 2017 Summary of all item comments reviewed. Authors 1, 2 and RA made *version T-3 for use in pre-testing II.*	
STEP 3 PRE-TESTING II	
HLS-EU-Q16-IS questionnaire: March 2017 An intended cross-sectional sample of 1200 people provided by the Icelandic national registry was sent an introduction letter, the HLS-EU-Q16-IS and 11 background questions by mail. The actual sample consisted of 251 individuals who completed the HLS-EU-Q16-IS. Data was analysed for internal consistency using Cronbach’s α, and construct validity was examined using exploratory factor analysis.	
Final version of the HLS-EU-Q16-IS	

#### Cognitive interviewing

The cognitive interviews took place at the workplace or at a given participant’s home, depending on their preference. The interviews lasted from 12 to 35 min (average – 25 min), were tape-recorded, and a written summary was made after each interview. Both the think-aloud interview method and the verbal-probing technique were used [20, 22]. The think-aloud part involved participants responding to the questionnaire in writing, while being asked to think out loud about what was going through their mind while responding to each item. This was to help the researcher capture participants’ understanding of each item, and to determine if they were struggling to understand the wording or comprehend the meaning of each item. As a follow-up to the think-aloud procedure, participants were also given verbal probes about: [1] their understanding and interpretation of specific items or ideas [2]; how they would rephrase items [3]; why they answered them the way they did; and [4], generally speaking, how easy or difficult it was to answer the items. After the first and second round of cognitive interviews, authors 1, 2 and the research assistant held a consultation meeting where summaries from the interviews were reviewed and the questionnaire changed accordingly. The goal of the second round of interviews was to get an opinion from individuals who had not seen the questionnaire, to confirm that there were no additional issues or comments on wording.

#### Validity testing of instrument

For the instrument validation, mode of data collection varied depending on the participant’s age. The intention was to maximise the response rate, by targeting each generation in the most user friendly way, yet within the surveys budget and timeframe. All intended participants were mailed an introduction letter along with the HLS-EU-Q16 Icelandic version and 11 background items. The background items were on age, gender, education, income and perception of own general health. For participants in the 18–64 age group (*n* = 990), the introduction letter included a QR code and a web URL so they could answer the questionnaire by computer-assisted (electronic) self-administration method. In the 65–85 age group (*n* = 210), the introduction letter included a questionnaire for a traditional paper and pencil self-administration and a prepaid response envelope. In addition, the information letter stated that they would receive a phone call from the researcher if the researcher had not received their answers in three weeks, so they would have the opportunity to answer the list by phone, a computer-assisted telephone interview. For that purpose, questionnaires for this age group were numbered. After four weeks, the response rate in the 18–64 years age group was low (< 20%) compared to over 60% in the 65–85 age group. Therefore, every fifth participant in the 18–64 age group was phoned and offered to answer the questionnaire by computer-assisted telephone interview or by computer-assisted (electronic) self-administration method (web-URL).

### Analysis of survey data

Descriptive statistics for the background of all survey participants (*n* = 251) included mean (M) and standard deviation (SD) for continuous variables and counts and proportions for categorical variables. Descriptive statistics were also used to present the preliminary norms on HLS-EU-Q16-IS by gender and age group.

A Principal Component Analysis (PCA) was conducted for structural analysis of the HLS-EU-Q16-IS, using an Oblimin rotation. In addition, a linear multivariate regression model was used to analyse the association between HL (dependent variable) and potential influencing factors (independent variables). The independent variables were selected for the model based on a significant bivariate correlation with HL. Statistical analyses were run with the IBM SPSS software package, v. 22 [27].

## Results

### Translation and adaptation based on cognitive interviewing

In the translation process, small differences were found in syntax and grammar between the forward and back translations of the questionnaire. The items were, however, semantically equivalent. The specialists´ committee reflected and reviewed translations and made critical decisions on “mental health” in item 8 and 13, “health warnings” in item 9 and “health screenings” in item 10 and about the role of pharmacist in different cultures connected to item four.

The translation process was followed by the first round of cognitive interviews with lay people including six women and six men, age range from 20 to 74 (*Mean* = 51 and *SD* ± 19.55) years. For the second round, four women and one man, age range 26 to 65 (*Mean* = 55 and *SD* ± 11.12) years participated. Eleven items of 16 in the HLS-EU-Q16-IS were changed after the two rounds of analyses following the cognitive interviews. The items appeared to become simpler and clearer as well as becoming more applicable to lay Icelandic language. After the analysis of responses, we also changed the appearance of the instructions for the questionnaire, switching the order of the responses to the scale’s items, starting with “very easy” instead of “very difficult”. Participants felt it important to have the most positive response the first one. A comprehensive overview of changes in the items of the questionnaire after the two rounds and consultation of researchers are shown in Table 2. As an example of minor changes in wording; in item 1, we replaced the medical term for “treatment” with a more general term which expresses wider variety of interventions. An example of a considerable change in wording is found in item 7, “… follow instructions from your doctor or pharmacist”. In the Icelandic version, the “pharmacist” was dropped because of cultural differences.

**Table 2:** Changes in wording of items due to cognitive interviewing

	Items with no changes	Items with minor changes	Items with considerable changes
First round	1, 2, 3, 4, 13, 14 and 15	5, 6, 10, 11, 12 and 16	7, 8 and 9
Second round	2, 3, 4, 5, 6, 7, 8, 11, 12, 13 and 15	1, 9, 10 and 14	16

### Survey participants

A total of 268 participants completed the survey form (response rate 24%), 119 on paper, 28 per phone and 121 per web URL. However, 17 participants had to be excluded because of more than two missing items on HLS-EU-Q16-IS. A total of 251 participants completed the questionnaire, age 18 to 85 years (*Mean* = 55, ±*SD* 18.98) and 52% women. The response rate was higher in the 65–85 age group compared to the 18–64 age group. Icelandic was the first language of 92.8% (233/251) of participants and the majority (72.5%; 182/251) lived in South Iceland, including the capital area. To gauge for potential hidden nesting effects due to response rates, sampling methods and geographic location of respondents, an intraclass coefficient (ICC) was calculated. The mean ICC for single measures was .322, indicating that the sample was not more homogeneous than if it had been drawn from a non-stratified sample. See Table 3 for characteristics of survey participants.

**Table 3:** Characteristics of the instrument testing sample (*n* = 251)

	Frequencies (n)	%
Age:		
18–39	55	21.9
40–59	52	20.7
60–85	137	54.6
Missing	7	2.8
Gender:		
Female	131	52
Male	115	46
Missing	5	2
Education level:		
Elementary	56	22.3
Secondary/Trade school	93	37
University degree	84	33.5
Other (e.g. professional pilot program, musical education)	7	2.8
Missing	11	4.4
Number of people in the household:		
1	45	17.9
2 or more	193	76.9
Missing	13	5.2
Income per month for the home after tax:		
< 1.440 EUR	14	5.6
1.440–2.885 EUR	87	34.6
> 2.885 EUR	134	53.4
Missing	16	6.4
Enough income after tax to fulfil needs:		
Yes	168	66.9
No	64	25.5
Missing	19	7.6
Self-rated health:		
Excellent	23	9.1
Very good	84	33.5
Good	64	25.5
Fair	53	21.1
Bad	16	6.4
Missing	11	4.4

### Validity testing of instrument

Table 4 shows responses to individual items by response options on the HLS-EU-Q16-IS, including missing values and skewness/kurtosis values for each item. Overall, missing values were few, the highest count of which was nine, on item 8.

**Table 4:** Responses to individual items on the HLS-EU-Q16-IS (*n* = 251)

Item nr.	Very easy	Fairly easy	Fairly difficult	Very difficult	Missing	Skewness	Kurtosis
1	70	141	32	7	1	.61	.53
2	108	110	25	5	3	.83	.42
3	139	99	8	1	4	.89	.50
4	190	60	0	0	1	1,22	−.50
5	63	108	68	9	3	.23	−.66
6	107	117	20	4	3	.81	.61
7	152	92	5	0	2	.77	−.59
8	76	107	49	10	9	.50	−.38
9	185	55	10	1	0	1.79	2,92
10	171	70	8	0	2	1.26	.62
11	59	92	77	17	6	.17	−.81
12	47	105	72	21	6	.23	−.61
13	106	111	25	4	5	.78	.30
14	106	108	29	3	5	.69	−.05
15	80	99	48	16	8	.57	−.45
16	166	73	7	2	3	1.55	2.62

Internal consistency for the HLS-EU-Q16-IS questionnaire was *α* = .88. The KMO value was .86, supporting the sampling adequacy for the analysis [28, 29]. Bartlett’s test of sphericity (*χ*^2^ = 1436.930, df = 120, *p* < 0.001) indicated that correlations in the correlation matrix did not occur by chance, and that correlation between items were sufficient for the analysis. The final data reduction model chosen for the current analysis was a Principal Component Analysis (PCA) with an Oblimin rotation. An initial analysis was run to obtain eigenvalues for each component in the data. Four components (eigenvalues 5.94, 1.62, 1.38 and 1.05) explained 62.6% of the variance. The scree plot indicated that the optimal solution consists of two to four components depending on how the plot is interpreted. The items´ loadings after rotation are illustrated in Table 5 with Cronbach’s alpha for each subscale. The items that cluster on the same factor suggest that component 1 (items: 3, 5, 6, 7 and *α* = .77) represents “Processing and Using Information from the Doctor”; component 2 (items: 11, 12, 14, 15 and *α* = .85) represents “Processing and Using Information from the Family and Media”; component 3 (items: 4, 9, 10, 13, 16 and *α* = .76) represents “Processing Information in Connection to Healthy Lifestyle”; and component 4 (items: 1, 2, 8 and *α* = .73) represents “Finding Information about Health Problems/Illnesses”. Component 1, “Processing and Using Information from the Doctor” had the highest internal consistency of the subscales that appeared in the current PCA. The corrected item-total correlation in each subscale was high as all items received correlation of .40 or higher, with a range from .47 to .85. Deleting items from subscales did not affect the internal consistency of the subscales.

**Table 5:** Component loadings of the HLS-EU-Q16-IS, after Oblimin rotation (*n* = 251)

Item	Component				*α*	Subscales
	1	2	3	4		
3. understand what your doctor says to you?	**.696**	−.075	−.016	.172	.77	Processing and Using Information from the Doctor
6. use information the doctor gives you to make decisions about your illness?	**.629**	−.267	−.020	.166		
7. follow instructions from your doctor?	**.626**	−.112	.219	−.136		
5. judge when may need to get a second opinion from another doctor?	**.503**	−.140	−.026	.376		
12. decide how you can protect yourself from illness based on information in the media?	−.019	**−.921**	−.067	.031	.85	Processing and Using Information from the Family and Media
11. judge if the information on health risks in the media is reliable?	−.004	**−.815**	.038	.059		
15. understand information in the media on how to get healthier?	.215	**−.801**	−.059	−.059		
14. understand advice on health from family members or friends?	.140	**−.505**	.269	.013		
16. judge which everyday behaviour is related to your health?	−.140	−.241	**.701**	−.043	.76	Processing Information in Connection to Healthy Lifestyle
9. understand health warnings about behaviour such as smoking, low physical activity and drinking too much?	.110	.112	**.685**	.009		
10. understand why you need health screenings?	.253	.004	**.621**	.022		
13. find out about activities that are good for your mental well-being?	−.229	−.345	**.531**	.367		
4. understand your doctor’s or pharmacist’s instructions on how to take a prescribed medicine?	.432	.130	**.457**	.120		
1. find information on treatments of illnesses that concern you?	.031	−.071	−.177	**.819**	.73	Finding Information about Health Problems/Illnesses
2. find out where to get professional help when you are ill?	.199	.137	.032	**.744**		
8. find information on how to manage mental health problems like stress or depression?	−.111	−.099	.278	**.715**		

*α* = Cronbach’s alpha

Loadings in bold represent items that loaded on each component

There were positive relationships among HL score and [1] education (*r* = .144, *p* = 0.037), [2] income per month (*r* = .167, *p* = 0.016), [3] having enough income after tax to fulfil needs (*r* = .205, *p* = 0.003), and a negative relationship between HL score and self-rated health (*r* = −.263, *p* = 0.001). No correlation between HL and age was found. A multivariate linear regression was conducted to analyse association between HL (dependent variable) and education level, income per month, having enough income to fulfil needs, and self-rated health (independent variables). A statistically significant regression relationship was found (*F* (4, 193) = 5.484, *p* < .001), with an *R*^*2*^ of .102. Self-rated health was the only statistically significant predictor of HL (*β* = −.484, *p* = .008.).

### Preliminary norms

Preliminary norms for HL were established in a general population of Icelanders. In this sample the scores ranged from 5 to 16, the mean was 13.7 (*SD* = ± 2.6) and the median 14. In Table 6, descriptive findings on HL score divided by gender and age groups are shown. There were 182 individuals (72.5%) that scored from 13 to 16 indicating sufficient HL, 55 (22%) scored from 9 to 12, which has been defined as problematic HL, and 14 (5.5%) scored from 0 to 8, indicating inadequate HL.

**Table 6:** HL score distribution within gender and age groups

Gender	Age	N	Mean (SD)	Median (min-max)
Women	18–39	30	13.48 (2.98)	15 (5–16)
	40–59	26	14.32 (2.13)	15 (8–16)
	60–85	74	13.74 (2.58)	14 (6–16)
Men	18–39	25	13.90 (2.13)	15 (9–16)
	40–59	26	13.84 (2.91)	15 (7–16)
	60–85	63	13.29 (2.59)	14 (6–16)

## Discussion

Our results indicate that, after translating and adapting the HLS-EU-Q16 to Icelandic, the HLS-EU-Q16-IS is a valid instrument, ready to be used in Iceland, and opening possibilities to study HL in Iceland and compare the results internationally.

In our view, to be consistent with the broad and inclusive definition of HL, we felt that lay people should be participants in the process. The cognitive interviewing using lay people was an important step in the translation process, which eventually led to the Icelandic version of HLS-EU-Q16. Although the specialist review turned out to be essential regarding accepted language within the health- and social setting, the cognitive interviewing gave vital information about the understanding of actual people who might answer the questionnaire. It also provided lay people an opportunity to influence the adaptation process. This was, for example, useful in the wording of items connected to culturally sensitive things, such as mental health and illness. Not only did the wording of items change with cognitive interviewing, but it also prompted the researchers to reverse the response scale, starting with “very easy” instead of “very difficult” as in the Swedish version [30]. Epstein et al. [16] has pointed out that currently there is no consensus on cross-cultural adaptation procedure. However, researchers suggest [24], the importance of including the target audience when translating questionnaires to another language.

The Icelandic version of HLS-EU-Q16 exhibited high internal consistency, with Cronbach’s *α* = .88, which is in line with results from the German version of the instrument [31]. The four components yielded by the PCA (Table 5) had internal consistency from *α* = .73 for component 4 to *α* = .85 for component 2. It should be noted that with a list of 16 items, lower internal consistency could be expected, so these results are quite acceptable. The PCA yielded a reasonably defined structure, and only item 4 loaded on more than one component (Table 5) ( “… understand your doctor’s or pharmacist’s instruction on how to take prescribed medicine?” loaded on components 1 and 3). This is possibly because participants perceived taking one’s prescribed medicine as a part of a healthy lifestyle, in addition to reflecting their interaction with the doctor. Another explanation could be that the word “pharmacist” was removed from item 7 in the Icelandic version but not in item 4. In the Icelandic culture, doctors and nurses play a dominant role in instructing people on how to take their medicine, whereas pharmacists play a negligible one.

In our principal component analysis, the fourth component includes items representing the competency to find health related information (Table 5). The other three components represented more than one competency in the original Sørensen’s et al. [10] model. This might indicate that finding health-related information is more salient for the Icelandic sample than, for example, understanding and appraising health-related information. The three domains of health care, disease prevention and health promotion are not indistinct in the current analysis. Component 1 includes only items within the health care domain and component 4 includes two of three items within that domain. Component 2 includes items from two domains and component 3 includes items within every three domains. This indicates subtle differences in how the Icelandic samples responds to those items and the original model. As previously reported, the HLS-EU-Q instrument has received critique for its continued emphasis on healthcare and disease prevention over health promotion [15]. The findings from our current PCA indicate that the four health promotion domain items do not cluster together as they did in the original version [10]. Considering the above, it is reasonable to conclude that the domains do not manifest in the same way across the cultures, comparing our study and the work of Sørensen et al. [10]. Our approach of using PCA seems relatively unique, yet, as PCA is an empirical technique, it should be well suited for analysis of cultural differences in item understanding of populations. While Confirmatory Factor Analysis seems more commonly used than a PCA or Exploratory Factor Analysis for validation of the HLS-EU-Q (e.g. [32–34]), exploratory techniques such as an EFA or PCA are nevertheless used in the field. For example, Sukys et al. [35] used an EFA to assess the factor structure of the healthcare, disease prevention, and health promotion domains for the HLS-EU-Q47.

The results from the current study demonstrated a relative high score in HL, compared to the European study [14], and limited HL was connected to people with lower education and income, while a negative correlation was seen between self-rated health as predictor of HL. People with lower education have been found to have lower health literacy in comparison to people with higher education [14, 36]. Interestingly, no negative correlation was discovered between age and HL in the current study, as previous studies from Europe, Australia and Asia have reported [14, 34, 37]. In a Danish national study [38], results indicated that people age 45–65 have less difficulty in understanding and engaging actively with healthcare providers, than those between the ages of 25 and 45. Attention is drawn to the necessity to look at HL also in connection to what kind of health care systems are provided in each country, and that, for example, Nordic counties with similar universal health care systems could more easily be compared together than counties with different systems. The current research is based on a sample of 251 people within a nation with approximately 350,000 inhabitants, a relatively large proportion compared to counties with larger populations. In addition, our sample was stratified according to age, gender and place of living and socio-demographic data of the respondents did not indicate difference from the general population. Therefore, our results may offer preliminary norms or benchmarks that can inform future surveys in countries with comparable health systems.

## Limitations

The low participation rate, 24%, (step three of the validation process of the instrument) is of some concern and a limitation to this study. However, the overall number of participants reached is acceptable within the frame of the statistical analysis used, and an intraclass correlation of .322 indicated that the assumption of independence was not violated. The reasons for the low participation rate can only be speculated upon, especially among people between the ages of 18 and 59. This age group had a participation rate of 40% (Table 3), compared to almost 55% in the 60–85 age group. We used diverse administration modes, a traditional paper and pencil self-administration by post, computer-assisted telephone interviews and computer-assisted electronic self-administration method (web URL). The impact of administration mode on response effects has been reported as well as the difficulty to separate out such effects [39, 40]. Although self-administered questionnaires are considered to have many benefits the mode has also been criticised for, among other things, to have a high cognitive burden and not offering additional explanations [39, 40]. The different administration modes in our study can be regarded as a limitation for between-studies comparison. However, by conducting cognitive interviews among lay people a step was taken to limit this effect.

Survey response rates have been declining over the past decade and there are indications that web-based questionnaires could be an alternative platform to reach higher participation rates in population surveys, compared to paper questionnaires [41]. An attempt was made to make answering more appealing or acceptable to people by offering a QR code. However, that option might be more used by younger people, under the age of 30.

## Conclusions

The findings indicated that the Icelandic version of HLS-EU-Q16 is psychometrically sound, with a reasonably clear factor structure, and comparable to the original model. This opens possibilities to study HL in Iceland, gradually add to a database, which now includes preliminary norms, and compare the results internationally. The specialist review and cognitive interviewing provided essential additional information to the translation-back-translation procedure. This translation model might be helpful for other countries where information on HL is missing based on lack of validated tools. We believe that this instrument will become valuable in a future cross-cultural research on HL among the public.

## Data Availability

The datasets used and/or analysed during the current study and the Icelandic version of the questionnaire are available from the corresponding author on reasonable request.

## Abbreviations

CCA: Cross-Cultural Adaptation
EC: European Commission
HL: Health literacy
HLS-EU-Q: The European Health Literacy Survey Questionnaire
HLS-EU-Q16: The European Health Literacy Survey Questionnaire- short version
ICC: Intraclass correlation coefficient
PCA: Principal Component Analysis
